# Larvicidal activity of antiparasitic plant extracts against ovine gastrointestinal nematodes: an *in vitro* study

**DOI:** 10.3389/fvets.2026.1805893

**Published:** 2026-05-12

**Authors:** Chao Ke, Niya Tu, Fatimat Shittu, Md Imranuzzaman, Dipsana Kc, Men Su, Thomas B. McFadden, Tumen Wuliji

**Affiliations:** 1 Division of Animal Science, College of Agriculture, Food and Natural Resources, University of Missouri, Columbia, MO, United States; 2 Department of Agriculture and Environmental Sciences, Lincoln University, Jefferson City, MO, United States

**Keywords:** anthelmintic resistance, anti-parasitic forage plants, *Haemonchus contortus*, larval motility assay, small ruminants

## Abstract

Increasing resistance of gastrointestinal nematodes (GINs) to synthetic anthelmintics undermines the sustainability of small ruminant production, underscoring the urgent need for alternative control strategies. This study evaluated the *in vitro* larvicidal activity of aqueous extracts from 24 plant species, including forages, common weeds, and herb species with reported antiparasitic properties. Third-stage larvae (L3) of sheep GINs (predominantly *Haemonchus contortus* and *Strongyloides papillosus*) were exposed to each aqueous extract at 500 or 750 μL/mL, and larval mortality was assessed at 12, 24, and 48-h post-exposure. Results showed a clear dose-dependent and time-dependent increase in larval mortality. At the higher dose, five extracts demonstrated ≥90% mortality of larvae at 48 h, including *Ageratum conyzoides*, *Euphorbia esula*, *Chenopodium album*, *Abutilon theophrasti*, and *Eupatorium altissimum*. Notably, *Ageratum conyzoides* and *Abutilon theophrasti* caused over 85% larval mortality within the first 12 h of exposure, and the high efficacy of plant *Eupatorium altissimum* and *Abutilon theophrasti* is a novel finding in this study. Principal component analysis (PCA) of efficacy data grouped the extracts into distinct high-, moderate, and low-efficacy tiers. High-efficacy species (e.g., *A. conyzoides*, *E. esula*, and *C. album*) clustered separately from moderate-efficacy species (e.g., *Solidago rugosa*, *Ocimum basilicum*, *Albizia julibrissin*) and low-efficacy species (e.g., *Cannabis sativa*, *Lespedeza cuneata*, *Plantago major*). Some efficacious plants also exhibited favorable nutritional profiles, for instance, *Dalea purpurea, Cichorium intybus, Lotus corniculatus*, and *Onobrychis viciifolia* combined antiparasitic effects with low fiber content and high *in vitro* digestibility. These results highlight that several plant species merit further evaluations as sustainable botanical dewormers for GIN control. This broad screening provides a foundation for *in vivo* trials and supports the future development of integrated parasite management strategies for small ruminant production.

## 1 Introduction

Gastrointestinal nematodes (GIN) are major health threats in small ruminant production, causing anemia, digestive disorders, and weight loss in infected animals. This diverse parasite group includes strongyle nematodes (e.g., *Haemonchus, Trichostrongylus, Teladorsagia*) and non-strongyles (*Strongyloides* spp), all of which impose substantial disease burdens in grazing systems (1). Among GINs, *Haemonchus contortus* is one of the most pathogenic species, causing severe anemia (2, 3), while *Strongyloides papillosus* contributes to productivity losses and is often underdiagnosed in the field (4). Collectively, GIN infections lead to poor weight gains, increased treatment costs, and higher mortality in small ruminants (5, 6). Historically, GIN control has relied on synthetic anthelmintic drugs (7). However, decades of frequent use of anthelmintics have led to widespread drug resistance, thereby reducing treatment efficacy and threatening the sustainability of small ruminant enterprises (8–10). Key drivers of resistance include over-reliance on anthelmintics, under-dosing, mass treatment strategies, and genetic adaptability of parasites (11). Anthelmintic resistance is now documented across all major drug classes, leaving producers with limited treatment options and necessitating more frequent dosing regimens to control GINs (12).

In response, integrated parasite management approaches, such as targeted selective treatments and refugia-based strategies, are being explored to slow the spread of resistance (13). Growing concern about anthelmintic resistance, coupled with consumer demand for residue-free animal products and sustainable livestock practices, has accelerated research into non-chemical and biologically-based control methods (14–16). One approach is the use of plant extracts that are rich in bioactive secondary metabolites, for example, condensed tannins, flavonoids, terpenoids, and alkaloids (17). Bioactive compounds exhibit diverse biological activities that can influence parasite survival. For example, certain phytochemicals disrupt GIN neuromuscular function or metabolism (18). Indeed, numerous tannin-rich forages and herbs have demonstrated antiparasitic properties (19). For instance, *Cichorium intybus* (chicory) and *Lotus corniculatus* (birdsfoot trefoil) are known to reduce GIN infection *in vitro* and *in vivo*, with sesquiterpene lactones and condensed tannins identified as key active components (20, 21). Similarly, *Ocimum basilicum* (basil) and *Ageratum conyzoides* (goatweed) contain phenolics, alkaloids, and other compounds that have demonstrated antiparasitic and antifungal activities (22, 23). However, direct anthelmintic efficacy against GIN has not been well documented for *Salvia azurea*, *Solidago rugosa*, *Dalea purpurea*, *Amaranthus spinosus*, or *Abutilon theophrasti*. Nevertheless, their phytochemical profiles suggest potential anthelmintic activity (24–26).

Moreover, previous studies often tested a narrow set of plant species with non-standard methodologies, which makes it difficult to compare plant species across studies. To address this gap, the present study evaluated a broad range of 24 plant species including forages, common weeds, and herbs under identical *in vitro* conditions to assess anthelmintic activity against ovine GIN larvae. These 24 plant species were selected to represent a diverse set of candidates with potential for on-farm use. Forage species were included due to prior evidence of high nutritional value and antiparasitic activity (27–29). Herb species were chosen based on their use in ethnoveterinary remedies against sheep parasites, and weed species were considered for their rich phytochemical profiles (30, 31). All plant extracts in this study were prepared using aqueous extraction to approximate on-farm preparation practices relevant to smallholder production systems.

Furthermore, we assessed the nutritional attributes of each plant, including *in vitro* true digestibility, neutral detergent fiber (NDF), acid detergent fiber (ADF), and crude fiber. Understanding these parameters integrates botanical anthelmintics into feeding programs on farms. Fiber composition is a key determinant of rumen function and digesta kinetics (32). Plants with high fiber content are associated with reduced ruminal passage rates, which may prolong gastrointestinal retention time and increase the exposure of parasites to bioactive compounds (33). In contrast, highly digestible forages (low fiber content) typically exhibit faster passage rate through the gastrointestinal tract, potentially limiting parasite exposure while supporting greater voluntary intake (34). By considering both nutritional value and anthelmintic activity, this study aims to identify plant resources that are not only effective against GIN but also could be utilized in organic smallholder systems through direct grazing, supplemental feed, or administration as herbal drenches. Our goal is to provide a basis for *in vivo* evaluation and further assessment of botanical extracts in parasite management frameworks.

## 2 Materials and methods

### 2.1 Plant collection and extract preparation

Twenty-four plant species (Table 1) were grown in a Lincoln University greenhouse (Jefferson City, MO; 38.58° N, 92.17° W). Plants were harvested in July 2023 during the daytime (10 a.m.-12 p.m.). Fresh aerial parts (cut from three inches above the soil) were harvested at flowering or mature vegetative stages. One exception is *Pachyrhizus erosus* from which leaves and seeds were collected separately to compare plant parts. Plant materials were immediately transported to the laboratory and stored in ziploc bags at −80 °C. Frozen samples were then freeze-dried for 48 h, at −85 °C using a FreeZone 6 L freeze dryer (Labconco, Fisher Scientific, Wal-tham, MA, United States) and subsequently milled through a 2-mm screen using a Thomas Wiley Mini Cutting Mill (Thomas Scientific, Swedesboro, NJ, United States).

**Table 1:** Scientific and common names of plant species evaluated for anthelmintic activity.^1^

Group^2^	Plant species	Common name	Family	Abbreviation^3^
Forage	*Cichorium intybus*	Chicory	*Asteraceae*	CC
	*Dalea purpurea*	Purple prairie clover	*Fabaceae*	PP
	*Lespedeza cuneata*	Sericea lespedeza	*Fabaceae*	LP
	*Lotus corniculatus*	Birdsfoot trefoil	*Fabaceae*	BT
	*Onobrychis viciifolia*	Sainfoin	*Fabaceae*	SF
	*Plantago lanceolata*	Narrowleaf plantain	*Plantaginaceae*	NL
Herb	*Artemisia annua*	Sweet wormwood	*Asteraceae*	SW
	*Cannabis sativa*	Hemp	*Cannabaceae*	HL
	*Ocimum basilicum*	Basil	*Lamiaceae*	BA
	*Pachyrhizus erosus*	Jicama^4^	*Fabaceae*	JB
	*Pachyrhizus erosus*	Jicama^5^	*Fabaceae*	JL
	*Salvia azurea*	Blue sage	*Lamiaceae*	BS
	*Urtica dioica*	Stinging nettle	*Urticaceae*	SN
Weed	*Abutilon theophrasti*	Velvetleaf	*Malvaceae*	VL
	*Ageratum conyzoides*	Goatweed	*Asteraceae*	GW
	*Albizia julibrissin*	Mimosa tree	*Fabaceae*	MT
	*Amaranthus spinosus*	Amaranth	*Amaranthaceae*	AL
	*Chenopodium album*	Goosefoot	*Amaranthaceae*	GF
	*Crotalaria juncea*	Sunn hemp	*Fabaceae*	SH
	*Eupatorium altissimum*	Tall boneset	*Asteraceae*	TB
	*Euphorbia esula*	Spurge weed	*Euphorbiaceae*	SP
	*Phytolacca americana*	American pokeweed	*Phytolaccaceae*	PW
	*Plantago major*	Broadleaf plantain	*Plantaginaceae*	BL
	*Solidago rugosa*	Goldenrod	*Asteraceae*	GR

^1^All are aerial parts of plants except Jicama.

^2^Group = general category of plants.

^3^Abbreviation = code for tables/figures.

^4^Jicama beans.

^5^Jicama leaves.

Aqueous extracts were prepared at a 1:10 (w/v) ratio, using 1 g of dried plant material per 10 mL of solvent. The ratio was chosen to approximate extract concentrations hypothetically achievable in the rumen based on sheep dry matter intake and rumen volume (35). For each plant, 3 g of dried ground sample was mixed with 30 mL of deionized water in a 50 mL conical tube (Nunc™, Thermo Fisher Scientific, Waltham, MA, United States). The mixture was vortexed for 5 min at 1000 rpm using a Digital Vortex Mixer (Fisher Scientific) and then incubated at room temperature for 24 h with intermittent shaking to facilitate extraction. After extraction, samples were centrifuged (Sorvall ST4 Plus, Thermo Scientific) at 3000 rpm for 10 min. The supernatants were then transferred and filtered through 0.45-μm syringe filters (Merck Millipore, Burlington, MA, United States). The aqueous plant extracts were stored in conical tubes at −20 °C until use. Before bioassays, extracts were thawed and gently mixed.

### 2.2 GIN larvae source and recovery

Mixed-species GIN were obtained from a donor flock of naturally infected *Katahdin* sheep grazing pastures comprising mainly fescues (Freeman Farm, Lincoln University of Missouri). The flock had received no anthelmintic treatment in the prior 6 months; however, formal resistance testing of the parasite population in the flock was not conducted. Fresh fecal samples were collected from pasture and individual fecal egg counts (FEC) were determined for each sample using a modified McMaster technique (36). Samples with FEC > 500 eggs per gram of feces (EPG) were selected, pooled, and homogenized for larval culture. For each replicate, approximately 30 g of pooled feces were placed in a 100-mm Petri dish (approximately ¾ full per dish; *n* = 10 dishes). To obtain infective third-stage larvae (L3), the pooled feces were incubated (Heratherm™ General Protocol Ovens, Thermo Fisher) at 27 °C for 13 days to allow eggs to hatch and develop. Larvae at the L3 stage were then recovered using the Baermann funnel technique (37). Fecal material was wrapped in cheesecloth, suspended in water so that motile larvae migrated out into the liquid and settled at the bottom of the funnel over 24 h. Larvae were collected and maintained in a larval suspension in deionized water at 25 °C for no more than 24 h before use in bioassays. Based on morphological identification of eggs and L3, the larval population was composed predominantly of *Strongyloides papillosus* (approximately 30%) and *Haemonchus contortus* (approximately 60%), with the remainder comprising other species.

### 2.3 *In vitro* larval mortality assay

Anthelmintic activity was assessed using a larval motility assay (LMA) as described by Demeler et al. (38). Approximately 150 infective third-stage larvae (L3) suspended in 500 μL of larval suspension were dispensed into each well of a 12-well flat-bottom plate. Plant extracts were then added to achieve final extract proportions of 500 μL/mL (v/v) (500 μL extract + 500 μL larval suspension; final volume 1.0 mL) or 750 μL/mL (v/v) (1.5 mL extract + 500 μL larval suspension; final volume 2.0 mL). Negative control wells received deionized water added to match the final volume of the corresponding treatment. Positive control wells received eprinomectin (Eprinex® pour-on, Merial), a water-soluble dewormer, selected to represent therapeutic exposure levels in small ruminants and expected to demonstrate mortality over 90%. Eprinomectin (5 mg/mL) was diluted in deionized water to working solutions of 0.4 or 1.6 mL product per 100 mL of deionized water. Thereafter, 500 μL of these solutions were mixed with 500 μL larval suspension in the assay wells, the final eprinomectin concentrations in the wells were 10 μg/mL and 40 μg/mL, respectively. Treatments and controls were randomized across plates to minimize positional effects. Seven replicates were conducted at each dose and time combination of each plant extract and assigned randomly for each replicate.

Larval motility was examined after 12, 24, and 48 h of incubation using a Nikon SMZ800 stereomicroscope (Marshall Scientific) at a working magnification range of 7.5× −63×. For each well, larvae were gently stimulated with a fine probe and observed for approximately 10–15 s to determine any movement. Larvae that remained immotile and exhibited a straight, rigid body posture after stimulation were considered dead. Mortality (%) was calculated as:


$$\begin{array}{l} \text{Mortality rate}\;(\%)=\\ \frac{\text{Number of dead larvae}}{\text{Total number of larvae}\;\mathrm{per}\;\text{well}\;\mathrm{at}\;\text{initial time point}}\times 100 \end{array}$$

### 2.4 Plant nutritional analysis

#### 2.4.1 *In vitro* true digestibility

*In vitro* true digestibility of 24 plant species was determined using the ANKOM DaisyII Incubator system (ANKOM Technology, Macedon, NY, USA) following the manufacturer’s protocol. Approximately 0.50 g of freeze-dried and ground plant samples (as described in 2.1) were weighed into ANKOM F57 filter bags, which were heat sealed before incubation. Rumen fluid was collected twice (June 2024 and August 2024) from two healthy cannulated cows under an ACUC-approved protocol at the Foremost Dairy Farm (Columbia, MO, United States). Donor cows were maintained on a fescue-based diet with a standard concentrate supplement prior to rumen fluid collection. Fresh rumen fluid was filtered through four layers of cheesecloth, placed in a pre-warmed insulated container with infusion of carbon dioxide, and transported immediately to the laboratory. The incubation medium consisted of rumen fluid mixed with buffer solution according to the ANKOM DaisyII protocol. Filter bags containing plant samples were incubated in digestion jars at 39 °C for 48 h under continuous rotation. After incubation, bags were removed, rinsed thoroughly with distilled water to remove microbial residues, and dried at 105 °C to a constant weight for determining residual dry matter. Each plant sample was analyzed in two independent runs with two replicates per run.

#### 2.4.2 Fiber analysis

Fiber composition of the 24 plant species was determined using an ANKOM 200 Fiber Analyzer (ANKOM Technology, Macedon, NY, United States), following operational procedure in the manual. Approximately 0.50 g of freeze-dried and ground plant samples (as described in 2.1) were weighed into ANKOM F57 filter bags for NDF and ADF analysis, while approximately 1 g of each sample were weighed for CF analysis. All sample bags were heat sealed prior to analysis. Neutral detergent fiber (NDF) was determined using the neutral detergent solution provided by ANKOM. Heat-stable *α*-amylase (4 mL) and sodium sulfite (0.5 g/50 mL of solution) were added into mixture. Detergent digestion process followed standard protocol for NDF. After digestion, bags were rinsed with acetone to remove residues. After acetone evaporation, sample bags were then dried at 105 °C to maintain constant weight for weighing. Acid detergent fiber (ADF) was determined using acid detergent solution according to the ANKOM operational procedures with the same downstream process as NDF analysis after detergent digestion process. Crude fiber (CF) was determined using ambient temperature acid (0.225 N H_2_SO_4_) and ambient temperature base solution (0.313 N NaOH) for digestion. After digestion and drying, ash correction was performed by combusting the residual material in a muffle furnace at 550 °C for 4 h to remove inorganic components. The residue (ash) remaining after digestion was used to calculate ash-corrected crude fiber content. All fiber analyses were conducted in duplicate for each plant sample, and results were expressed on a dry matter basis.

### 2.5 Statistical analysis

Larval mortality data were compiled as mean ± standard deviation (SD) for each treatment, time, and concentration. A 2 × 3 factorial analysis was used to test the effects of extract concentration 50% vs. 75% v/v (equivalent to 500 μL/mL vs. 750 μL/mL), exposure time (12, 24, 48 h), and their interaction on larval mortality rate. Tukey’s Honestly Significant Difference (HSD) post-hoc test was applied for pairwise comparisons. Dunnett’s tests were conducted to compare all plant extracts with negative control and positive control. In addition, a principal component analysis (PCA) was performed on the mortality dataset (24 plants × 6 conditions (2 concentrations × 3 times)) to visualize clustering patterns of plant extract efficacy. All analyses were conducted with R 4.2.2 statistics (R Foundation for Statistical Computing, Vienna, Austria) using RStudio, with the STATS and FactoMineR packages for ANOVA and PCA, respectively. The threshold for statistical significance was set at *α* = 0.05 for all tests.

## 3 Results

### 3.1 Temporal progression of cumulative larvicidal activity

Larval mortality differed among plant species, extract concentration, and exposure duration (Table 2). Across most treatments, mortality increased with both concentration and incubation time, with the highest mortality observed at 48 h.

**Table 2:** Mean cumulative larval mortality (%) for each plant extract treatment at 12 h, 24 h, and 48 h of incubation.

Group	Treatments	Volume	Cumulative mortality^1^ (%) at 12 h	Cumulative mortality (%) at 24 h	Cumulative mortality (%) at 48 h
Forage	*Cichorium intybus*	750 μL/mL	40.2 (±16.0) bcd	74.7 (±14.3) def	76.6 (±14.7) bc
		500 μL/mL	22.6 (±20.0) abcd	44.0 (±23.4) bcde	64.9 (±23.6) bcd
	*Dalea purpurea*	750 μL/mL	68.2 (±10.3) defgh	83.0 (±10.7) ef	82.7 (±8.0) bc
		500 μL/mL	38.5 (±8.0) bcdefg	69.1 (±14.0) cdefg	78.8 (±14.7) bcd
	*Lespedeza cuneata*	750 μL/mL	31.3 (±14.6) abc	33.2 (±9.7) ab	70.9 (±13.8) bc
		500 μL/mL	32.28 (±16.9) abcdef	37.51 (±9.9) b	56.0 (±6.2) b
	*Lotus corniculatus*	750 μL/mL	58.95 (±28.2) cdefgh	66.3 (±5.7) cdef	77.5 (±10.2) bc
		500 μL/mL	56.10 (±23.3) efghi	62.0 (±10.0) bcdef	64.2 (±10.0) bcd
	*Onobrychis viciifolia*	750 μL/mL	41.6 (±18.0) bcde	50.7 (±18.9) bcd	76.6 (±10.2) bc
		500 μL/mL	29.9 (±5.7) abcdef	42.8 (±17.8) bcd	67.4 (±14.2) bcd
	*Plantago lanceolata*	750 μL/mL	45.7 (±11.1) bcdef	61.9 (±10.3) bcdef	81.0 (±9.3) bc
		500 μL/mL	42.7 (±8.8) bcdefgh	52.2 (±7.4) bcdef	54.9 (±8.9) b
Herb	*Artemisia annua*	750 μL/mL	58.0 (±13.7) bcdefg	67.8 (±8.0) cdef	79.5 (±6.5) bc
		500 μL/mL	31.6 (±19.4) abcdef	39.5 (±11.3) bc	62.7 (±8.1) bcd
	*Cannabis sativa*	750 μL/mL	45.9 (±11.6) bcdef	61.90 (±11.9) bcdef	73.8 (±22.7) bc
		500 μL/mL	43.3 (±12.5) bcdefgh	47.8 (±9.4) bcde	56.9 (±16.4) bc
	*Ocimum basilicum*	750 μL/mL	47.0 (±2.2) bcdef	75.5 (±12.1) def	83.7 (±10.8) bc
		500 μL/mL	36.9 (±17.6) bcdefg	53.9 (±25.3) bcdefg	63.8 (±28.8) bcd
	*Pachyrhizus erosus* (B)	750 μL/mL	62.7 (±29.8) defgh	81.3 (±12.0) ef	91.7 (±6.1) bc
		500 μL/mL	26.4 (±24.2) abcde	49.0 (±28.7) bcde	69.3 (±32.7) bcd
	*Pachyrhizus erosus* (L)	750 μL/mL	59.1 (±17.6) cdefgh	76.3 (±5.3) def	81.3 (±8.8) bc
		500 μL/mL	50.4 (±9.2) defghi	55.0 (±15.2) bcdefg	61.5 (±12.6) bcd
	*Salvia azurea*	750 μL/mL	40.9 (±31.2) bcd	64.6 (±21.1) cdef	82.9 (±6.9) bc
		500 μL/mL	20.5 (±12.6) abc	46.9 (±13.6) bcde	71.5 (±18.8) bcd
	*Urtica dioica*	750 μL/mL	85.8 (±12.7) gh	89.1 (±4.4) ef	89.0 (±3.2) bc
		500 μL/mL	69.8 (±16.0) hi	73.8 (±10.1) efg	81.4 (±13.0) bcd
Weed	*Abutilon theophrasti*	750 μL/mL	88.0 (±7.6) h	90.5 (±7.6) f	95.3 (±3.9) c
		500 μL/mL	69.5 (±11.9) hi	81.1 (±9.8) fg	86.7 (±13.8) bc
	*Ageratum conyzoides*	750 μL/mL	87.2 (±11.4) h	90.5 (±3.5) ef	95.3 (±3.6) bc
		500 μL/mL	68.0 (±16.4) defgh	80.1 (±9.4) fg	87.0 (±7.2) bc
	*Albizia julibrissin*	750 μL/mL	28.8 (±12.4) ab	60.4 (±7.3) bcde	87.6 (±7.0) bc
		500 μL/mL	17.4 (±8.8) ab	35.4 (±16.1) ab	57.0 (±12.7) bc
	*Amaranthus spinosus*	750 μL/mL	53.2 (±16.8) fgh	57.6 (±6.0) ef	70.2 (±4.5) bc
		500 μL/mL	46.6 (±30.0) bcdefghi	48.6 (±23.8) bcde	58.2 (±31.7) bcd
	*Chenopodium album*	750 μL/mL	69.1 (±12.0) defgh	81.3 (±6.5) ef	90.18 (±6.4) bc
		500 μL/mL	57.0 (±12.9) fghi	67.9 (±14.0) cdefg	78.8 (±8.0) bcd
	*Crotalaria juncea*	750 μL/mL	66.9 (±11.2) defgh	81.7 (±11.4) ef	88.2 (±8.2) bc
		500 μL/mL	63.1 (±11.6) ghi	70.4 (±11.9) defg	75.1 (±13.4) bc
	*Eupatorium altissimum*	750 μL/mL	70.8 (±6.7) efgh	86.8 (±9.8) ef	90.5 (±5.9) c
		500 μL/mL	35.1 (±14.6) bcdefg	60.7 (±6.1) bcdefg	74.2 (±10.6) bcd
	*Euphorbia esula*	750 μL/mL	70.7 (±23.5) efgh	86.4 (±17.1) ef	94.6 (±14.6) c
		500 μL/mL	35.2 (±24.9) abcdef	67.0 (±28.0) cdefg	76.3 (±36.9) bc
	*Phytolacca americana*	750 μL/mL	28.5 (±17.4) ab	39.4 (±30.8) bc	75.1 (±13.6) bc
		500 μL/mL	20.2 (±14.2) abc	35.6 (±28.1) ab	60.3 (±21.3) bcd
	*Plantago major L*	750 μL/mL	63.6 (±30.3) defgh	79.0 (±15.0) def	88.7 (±8.2) bc
		500 μL/mL	50.6 (±16.8) defghi	62.5 (±19.5) bcdefg	71.1 (±21.3) bcd
	*Solidago rugosa*	750 μL/mL	79.8 (±4.1) gh	90.0 (±7.0) ef	88.7 (±7.3) bc
		500 μL/mL	50.5 (±27.3) defghi	71.8 (±6.2) defg	81.4 (±4.4) bcd

^1^Cumulative mortality is presented as mean ± standard deviation (SD). Different lowercase letters within the same column indicate statistically significant differences among treatments, as determined by one-way analysis of variance (ANOVA) followed by Tukey’s honestly significant difference (HSD) test at *p* < 0.05. (Abbreviations of plant names are defined in Table 1).

Extracts of several plant species exhibited time-dependent larvicidal activity at high concentration (750 μL/mL). *Cichorium intybus* demonstrated mortality from 40.16 ± 15.98% at 12 h to 76.61 ± 14.72% at 48 h. A similar pattern was observed for *Dalea purpurea*, with mortality increasing from 68.18 ± 10.31% at 12 h to 82.67 ± 7.98% at 48 h. In *Lespedeza cuneata*, mortality increased from 31.30 ± 14.63% at 12 h to 70.91 ± 13.81% at 48 h. At the low concentration (500 μL/mL), larvicidal activity was lower than high concentration extracts for most species but followed similar temporal trends. For example, *Cichorium intybus* increased from 22.64 ± 19.89% at 12 h to 64.92 ± 23.55% at 48 h, while *Dalea purpurea* increased from 38.48 ± 8.00% to 78.84 ± 14.73% over the same period.

Several weed and herb species exhibited high larval mortality by 48 h at high concentration. *Ageratum conyzoides* showed the highest mortality (95.34 ± 3.62%), followed by *Abutilon theophrasti* (95.31 ± 3.9), *Euphorbia esula* (94.59 ± 4.71%), *Chenopodium album* (90.18 ± 6.44%), *Eupatorium altissimum* (90.54 ± 5.88%), *Solidago rugosa* (88.73 ± 7.26), *Crotalaria juncea* (88.16 ± 8.21%), *Albizia julibrissin* (87.63 ± 6.97%). At low concentration, mortality values for these species were lower compared to high concentration extracts but remained higher than other treatments.

### 3.2 Comparison with control treatments

All plant extracts resulted in significantly higher larval mortality than the negative control (water), which averaged 3.62 ± 1.27%, 5.80 ± 0.93%, and 5.73 ± 0.73% at 12, 24 and 48 h, respectively. Comparison of overall larvicidal activity of each plant extract (i.e., averaged over all time points and concentrations) to the average of the water control confirmed highly significant differences in all cases (Dunnett’s test, *p* < 0.001 for each extract vs. control). Dunnett’s analysis also revealed that most extracts had significantly (adjusted p < 0.001) lower efficacy than the commercial anthelmintic positive control (Table 3). However, a few plant treatments achieved mortality similar to the drug, in particular, *Ageratum conyzoides*, *Urtica dioica*, and *Abutilon theophrasti* showed no statistically significant difference in mortality compared to EP (*p* = 0.99, *p* = 0.38 and *p* = 1.00, respectively).

**Table 3:** Larval mortality: Dunnett’s multiple comparisons^1^ of overall average larvicidal activity of each plant extract vs. positive control (EP, eprinomectin).

Comparison	Difference in mean mortality	95% CI	*t*	*p* (adj., Dunnett)	Sig^2^
AL—EP	−48.5	[−56.6, −40.40]	−17.8	<0.001	***
BA—EP	−34.1	[−42.2, −26.0]	−12.5	<0.001	***
BL—EP	−52.0	[−60.1, −43.9]	−19.0	<0.001	***
BS—EP	−36.8	[−45.0, −28.7]	−13.5	<0.001	***
BT—EP	−25.8	[−33.9, −17.7]	−9.4	<0.001	***
CC—EP	−42.2	[−50.3, −34.1]	−15.4	<0.001	***
CT—EP	−78.0	[−86.1, −69.9]	−28.6	<0.001	***
GF—EP	−26.5	[−34.6, −18.4]	−9.7	<0.001	***
GR—EP	−17.5	[−25.6, −9.4]	−6.4	<0.001	***
GW—EP	2.1	[−6.0, 10.2]	0.8	0.9999	
HL—EP	−33.8	[−41.9, −25.7]	−12.4	<0.001	***
JB—EP	−28.8	[−36.9, −20.7]	−10.5	<0.001	***
JL—EP	−19.1	[−27.2, −11.0]	−7.0	<0.001	***
LP—EP	−40.7	[−48.8, −32.6]	−14.9	<0.001	***
MT—EP	−16.8	[−24.9, −8.6]	−6.1	<0.001	***
NL—EP	−30.5	[−38.6, −22.4]	−11.2	<0.001	***
PP—EP	−16.6	[−24.7, −8.5]	−6.1	<0.001	***
PW—EP	−47.1	[−55.2, −39.0]	−17.3	<0.001	***
SF—EP	−34.9	[−43.0, −26.8]	−12.8	<0.001	***
SH—EP	−11.0	[−19.1, −2.9]	−4.0	0.0013	**
SN—EP	−5.7	[−13.8, 2.4]	−2.1	0.3786	
SP—EP	−12.7	[−20.8, −4.6]	−4.7	<0.001	***
SW—EP	−36.8	[−44.9, −28.7]	−13.5	<0.001	***
TB—EP	−8.9	[−17.0, −0.8]	−3.3	0.0209	*
VL—EP	−1.6	[−9.7, 6.5]	−0.6	1.0000	

^1^Comparison of overall mean mortality (across all times and concentrations) for each extract to mean mortality for the positive control, eprinomectin (EP).

^2^Significance of comparisons: ***p < 0.001, **p < 0.01, *p < 0.05, *n* = 7 per extract-time combination.

### 3.3 Principal component analysis (PCA)

**Figure 1:**
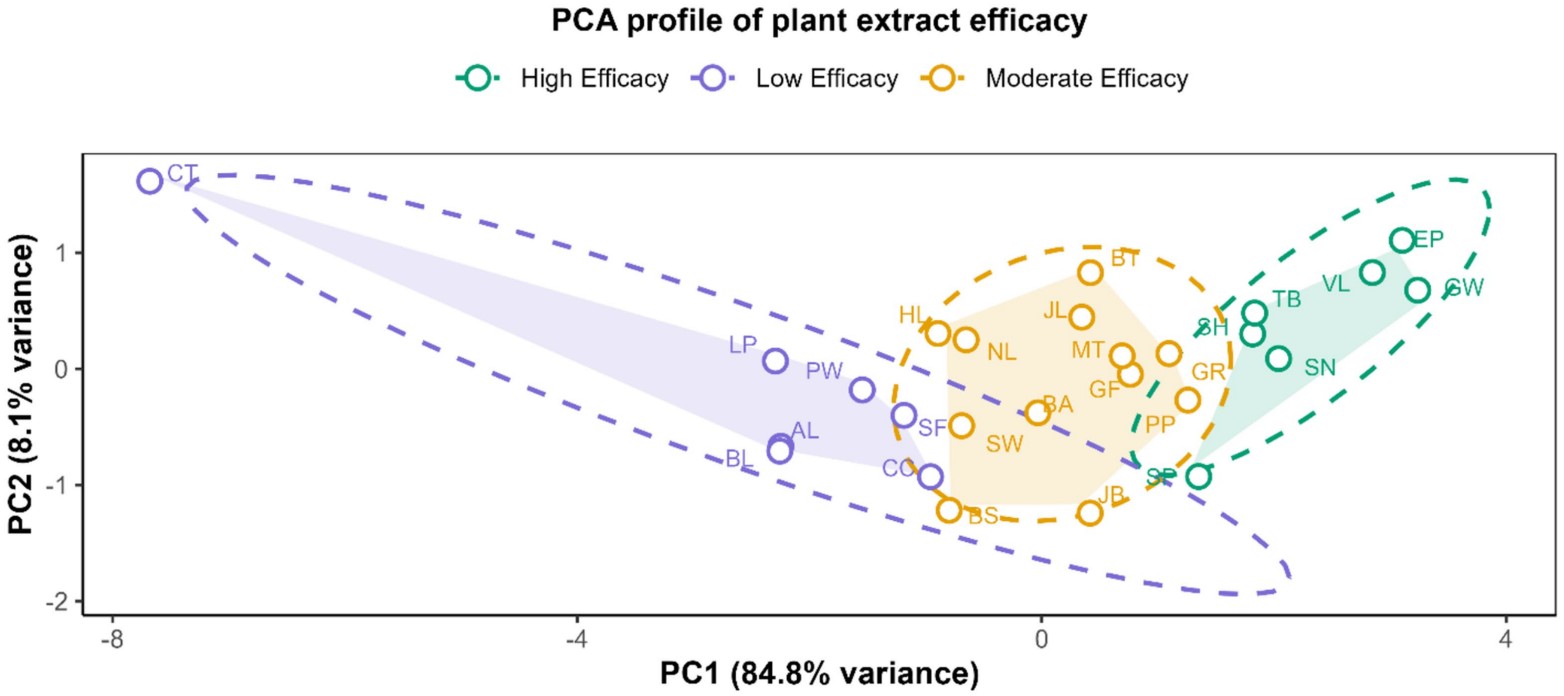
PCA plot of larvicidal efficacy profiles of plant extracts. Plant species and corresponding abbreviations used in the figure are: *Amaranthus spinosus* (AL), *Ocimum basilicum* (BA), *Plantago major* L (BL), *Salvia azurea* (BS), *Lotus corniculatus* (BT), *Cichorium intybus* (CC), *Chenopodium album* (GF), *Solidago rugosa* (GR), *Ageratum conyzoides* (GW), *Cannabis sativa* (HL), *Pachyrhizus erosus* (Bean) (JB), *Pachyrhizus erosus* (Leaves) (JL), *Lespedeza cuneata* (LP), *Albizia julibrissin* (MT), *Plantago lanceolata* (NL), *Dalea purpurea* (PP), *Phytolacca americana* (PW), *Onobrychis viciifolia* (SF), *Crotalaria juncea* (SH), *Urtica dioica* (SN), *Euphorbia esula* (SP), *Artemisia annua* (SW), *Eupatorium altissimum* (TB), and *Abutilon theophrasti* (VL). Additionally, control groups: negative control (CT) and positive control (EP).

Principal component analysis captured 93.4% of the variance (PC1: 84.8%; PC2: 8.1%) (Figure 1). Based on the distribution of mean mortality rates, overall mean extract efficacy was classified into three tiers: high efficacy (≥75th percentile; here ≥70% mortality), moderate efficacy (25th–75th percentile; 54%–70%), and low efficacy (≤25th percentile; ≤54% mortality). The PCA results revealed clustering of extracts by efficacy tier. High-efficacy extracts (e.g., *A. conyzoides*, *E. esula*, *C. album*, *Abutilon theophrasti*, *Eupatorium altissimum*) are grouped toward the positive end of PC1, indicating higher larvicidal effect. Moderate-efficacy extracts (e.g., *Solidago rugosa*, *Urtica dioica*, *Albizia julibrissin*, *Crotalaria juncea*, *Ocimum basilicum*) lie in the intermediate region of the PCA space. Low-efficacy extracts (e.g., *Cannabis sativa*, *Lespedeza cuneata*, *Phytolacca americana*, *Plantago major* and negative control) cluster toward the negative end of PC1, indicating lower larvicidal effect.

### 3.4 Nutritional value analysis and *in vitro* true digestibility

To assess the suitability of each of these plants as a potential feed source for small ruminants, whether by grazing or fed as hay or a pelleted or ground supplement, fiber content and digestibility were determined (Table 4). The plants are listed by descending IVTD for ease of comparison.

**Table 4:** Fiber composition and *in vitro* true digestibility (IVTD) of tested plant samples.

Common name	Abbreviation	ADF (%)	NDF (%)	CF (%)	IVTD (%)^1^
Hemp leaves	HL	32.1	39.4	16.1	93.6 ± 0.7a
Chicory	CC	24.9	30.7	15.3	92.9 ± 1.8a
Stinging nettle	SN	22.9	34.3	10.8	91.3 ± 0.3a
Jicama (leaves)	JL	35.2	43.8	19.1	91.3 ± 0.8a
Goosefoot	GF	28.4	39.7	19.7	91.3 ± 0.6a
Sweet wormwood	SW	18.3	30.7	12.9	90.0 ± 1.3ab
Broadleaf plantain	BL	30.3	38.6	13.5	86.4 ± 1.0bc
American pokeweed	PW	18.6	27.5	15.5	85.8 ± 0.6bc
Basil	BA	17.5	29.3	7.1	85.5 ± 0.8c
Birdsfoot trefoil	BT	38.2	40.2	18.1	85.4 ± 3.4c
Velvetleaf	VL	17.6	27.9	8.3	84.4 ± 0.5 cd
Amaranth	AL	19.2	19.6	15.5	84.0 ± 1.3 cd
Jicama (beans)	JB	25.3	34.8	13.9	83.2 ± 1.1d
Sunn hemp	SH	23.5	37.5	19.1	81.0 ± 2.5e
Tall boneset	TB	17.6	24.0	10.4	79.7 ± 1.2f
Purple prairie clover	PP	30.8	44.8	23.8	79.5 ± 0.7f
Sainfoin	SF	34.8	40.7	19.4	77.0 ± 2.2 g
Blue sage	BS	26.5	33.6	13.7	75.8 ± 1.6gh
Narrowleaf plantain	NL	21.1	40.7	13.3	75.4 ± 1.7gh
Mimosa tree	MT	31.4	44.8	19.9	75.0 ± 0.5 h
Goldenrod	GR	20.6	25.6	12.7	72.3 ± 2.0i
Goat weed	GW	18.8	34.4	10.4	70.0 ± 3.0j
Sericea lespedeza	LP	30.1	54.0	20.3	69.1 ± 1.8jk
Spurge weed	SP	15.6	38.7	16.0	68.2 ± 1.9 k

^1^IVTD: Mean and standard deviation of in vitro true digestibility. Letters indicate significant differences between samples (a > b > c …) for IVTD based on Tukey’s HSD test). All values on dry matter basis.

IVTD ranged from 93.6 ± 0.7% in hemp leaves (highest) down to 68.2 ± 1.9% in spurge weed (*E. esula*, lowest). There was considerable variation in NDF, ADF, CF, and IVTD among the 24 plant species (Table 4). The three most digestible plants were hemp leaves (IVTD 93.60 ± 0.73%), chicory (92.87 ± 1.75%), and stinging nettle (91.31 ± 0.27%). Notably, these species also had low fiber contents: for example, hemp leaves (ADF 32.1, NDF 39.4, CF 16.09) and chicory (ADF 24.91, NDF 30.65, CF 15.33). Such low fiber levels correspond with high IVTD, indicating these plants are highly digestible forages that would likely have a rapid rate of passage from the rumen, potentially affecting exposure, *in vivo*. Conversely, species with higher fiber content, notably sericea lespedeza, purple prairie clover, and mimosa tree demonstrated lower IVTD values. Overall, IVTD values showed an inverse association with fiber content across plant species.

### 3.5 Comparison by plant groups

**Figure 2:**
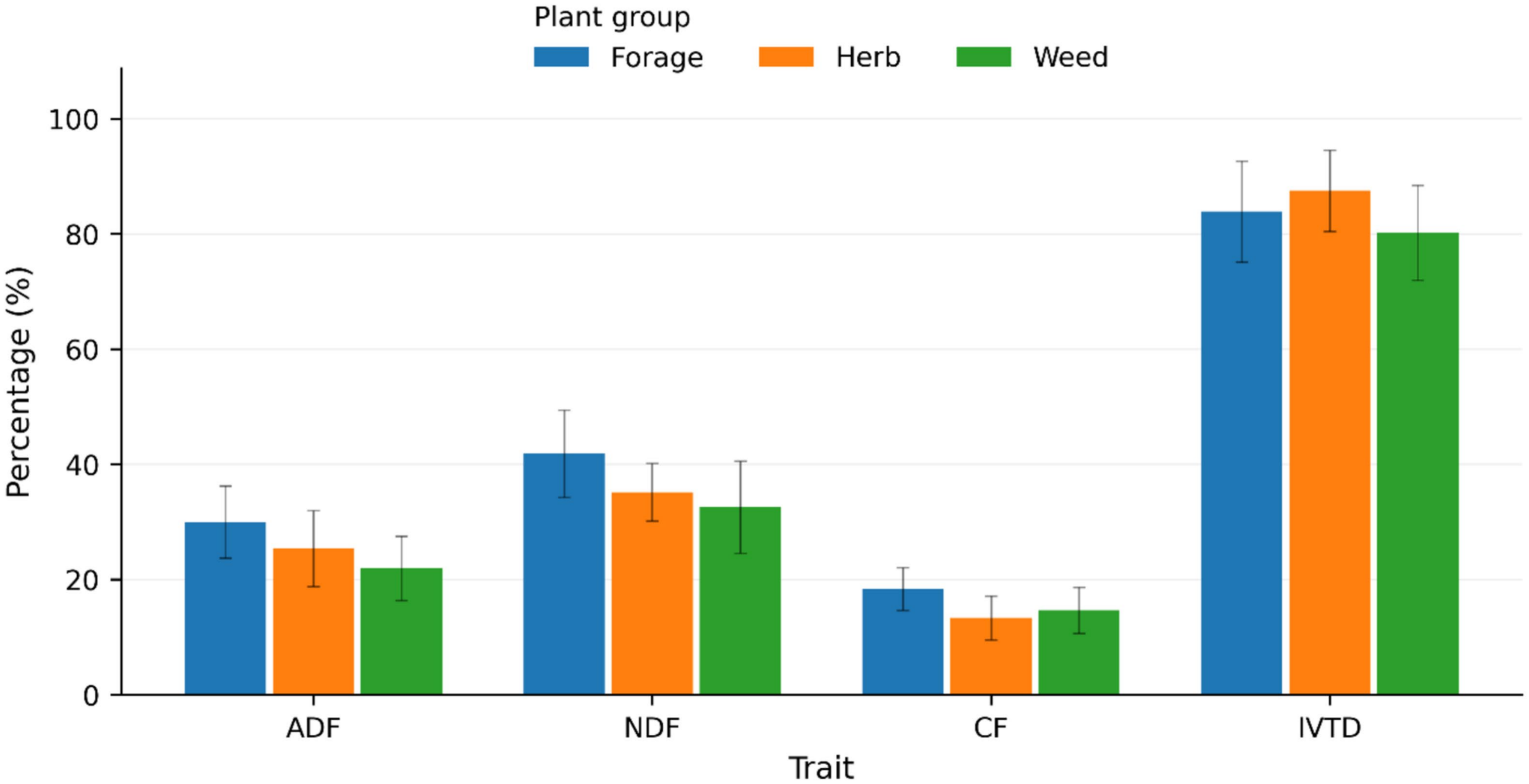
Comparison of ADF, NDF, CF, and IVTD (trait) among the plants, grouped as forages, herbs, or weeds.

The herbs contained lower ADF and NDF compared to forages, indicating higher potential digestibility (Figure 2). Forages contained lower CF than Herbs, suggesting a compositional difference in fiber type, but overall, herbs demonstrated higher IVTD values than forages (Figure 2). Weeds contained significantly (*p* < 0.05) lower ADF and NDF than forages, but higher IVTD (Figure 2). In comparing herbs to weeds, herbs had less ADF, NDF, and CF compared to weeds, consistent with herbs being the most digestible group overall.

## 4 Discussion

### 4.1 Summary of principal findings

The objective of this study was to conduct a broad comparative screening of potentially antiparasitic plants under uniform *in vitro* conditions. Out of 24 species tested, we identified five plant extracts: *Ageratum conyzoides, Euphorbia esula, Chenopodium album, Eupatorium altissimum*, and *Abutilon theophrasti* that consistently caused very high larval mortality (≥ 88% kill at 750 μL /mL, 48 h). In contrast, other plants showed only moderate or low activity, such as *Lespedeza cuneata, Cannabis sativa*, and *Phytolacca americana*, which demonstrated mortality rates below 70%. It is noteworthy that several plants which had not been previously reported for antiparasitic effect of GIN, for example, *Eupatorium altissimum* and *Abutilon theophrasti*, demonstrated high antiparasitic efficiency in the present study.

### 4.2 Comparative efficacy of extracts relative to prior findings

Plant larvicidal efficiency rankings generally aligned with scattered findings from prior research. For example, *A. conyzoides* and *E. esula* have been highlighted for strong antiparasitic activity, and our data support that these genera are among the most efficacious (39). Likewise, *E. esula*, in our study, agreed with the known anthelmintic properties of *Euphorbia hirta* (a relative of *E. esula*) that was reported to cause 100% mortality of adult *H. contortus* worms at 10 mg/mL within 6 h in a previous study (40). In our results, *E. esula* caused 75% mortality of L3 by 12 h and 94% by 48 h, which is comparable to Dicko’s study considering the different concentration (10 mg/mL vs. 750 μL /mL) and life stage (L3 vs. adult stage). Though comparisons between studies require careful interpretation given the variability in experimental endpoints (larvae stages) and the use of distinct parasite strains, those prior reports provide support for the efficacy observed in the present study. On the other hand, certain discrepancies between our results and *in vivo* studies highlight important considerations. We found that *Lespedeza cuneata*, a tannin-rich forage known to reduce parasite fecal egg counts in grazing animals, showed only moderate larvicidal activity in our study (71% mortality), even though feeding trials in sheep and goats have shown that fresh *Lespedeza cuneata* can significantly reduce fecal egg counts (41). Our findings provide first-time efficacy data on several species. The high efficacy of *Eupatorium altissimum* is a novel finding in this study. Similarly, *Abutilon theophrasti* showed strong larvicidal activity. While *Abutilon* species have documented ethnomedicinal uses and contain flavonoids and terpenoids, this is the first report, to our knowledge, of *A. theophrasti* exhibiting anthelmintic efficacy (42, 43).

### 4.3 Mechanisms of anthelmintic action and phytochemical rationale

The high larvicidal activity of *Ageratum conyzoides, Chenopodium album, Abutilon theophrasti,* and *Euphorbia esula* may be consistent with their phytochemical profiles and properties. *Ageratum conyzoides*, for example, contains chromenes (e.g., ageratochromene) and flavonoids that can disrupt nematode neuromuscular function (44). *Chenopodium album* is rich in ascaridole, a monoterpene peroxide known to induce oxidative stress, membrane destabilization, and apoptosis in helminths (45–48). Correspondingly, *Euphorbia esula* demonstrated efficacy with mortality rates approaching 95%, aligning with a neurotoxic mechanism of action likely mediated by bioactive constituents, for example, diterpenes within the plant’s latex (49). *Abutilon theophrasti* contains triterpenoids, saponins, and flavonoids among other constituents, indicating a combined anthelmintic action (50).

### 4.4 Aqueous extracts and on-farm feasibility

Many prior phytochemical studies have used organic solvents to extract lipophilic compounds (51, 52). However, use of organic-solvent extraction protocols limits the applicability of these approaches in smallholder farming systems. In contrast, aqueous extracts can more closely mimic on-farm usage, for example, application of botanical drenches. Moreover, that aqueous extracts of *Ageratum conyzoides, Chenopodium album,* and *Euphorbia esula* had high antiparasitic efficiency demonstrates that their active components, in this study, is water-soluble. These results serve as proof-of-concept that farmers could utilize water-based extracts as crude botanical preparations (for example, as a drench) that maintain high efficacy without the expense and chemical hazards of a solvent-based extraction process. The feasibility of application is further supported by our nutritional analysis of the tested plants. Based on our fiber and IVTD analysis, some of the efficacious plants also have favorable nutritional profiles. For example, bioactive forage species, including *Dalea purpurea*, *Cichorium intybus*, *Lotus corniculatus*, and *Onobrychis viciifolia* could offer a multifunctional approach to farming by serving as highly digestible feed sources that also function as natural antiparasitic agents.

### 4.5 Limitations and constraints

It must be emphasized that *in vitro* results may not accurately predict *in vivo* outcomes. In a living animal, factors such as compound metabolism, absorption, and interactions with the gut microbiome can influence efficacy and safety of plant-based treatments (53). Some plants that showed only moderate efficacy in our assay might still confer benefits in animals through longer-term exposure or indirect effects (e.g., *Lespedeza cuneata*) (54). Conversely, plants that were highly effective *in vitro* require confirmation *in vivo* to determine if they can achieve adequate concentrations in the gastrointestinal tract without causing toxicity to the host (55). Another limitation is the short duration and specific life stage of worms used in our bioassay. We measured larval mortality within 48 h against third-stage larvae (L3) of nematodes. In the actual host, parasites go through multiple life stages (eggs, L1-L2 larvae in the environment, L3 infective stage, and then L4 and adult worms in the host) (56). Thus, the acute lethality in our study favors compounds with quick-acting effects on larvae, potentially underrepresenting plants that have prolonged activities or preferentially affect other GIN life stages. A further potential limitation is the palatability of the plants. This could affect application, for example being grazed or fed as hay, but those constraints could be overcome by administration as a drench or by compounding into a pellet containing highly palatable ingredients. Examination of palatability was deemed beyond the scope of the present study.

### 4.6 Future work

The ultimate test of any anthelmintic is its performance in the target host. Thus, the next step will be to evaluate the top-performing plant candidates in animal trials. It will also be important to explore formulation and delivery methods for these plants. While farmers can use crude preparations, there may be benefits to creating more user-friendly forms, for example, concentrated powders, pelleted supplements, or encapsulated extracts that protect active compounds through the application. Parallel to animal trials, plant phytochemical investigations can identify and characterize the active metabolites responsible for anthelmintic effects. High-performance liquid chromatography coupled to mass spectrometry (HPLC-MS) and bioassay-guided experimental design can be employed on the most effective extracts.

## 5 Conclusion

Our *in vitro* bioassay demonstrates that aqueous extracts from diverse plant species can possess strong larvicidal activity against ovine gastrointestinal nematodes. Among the 24 species tested, *Ageratum conyzoides, Euphorbia esula, Chenopodium album, Abutilon theophrasti, and Eupatorium altissimum* demonstrated the greatest larval mortality, whereas others showed moderate (e.g., *Solidago rugosa*, *Ocimum basilicum*, *Albizia julibrissin*) or low effects (e.g., *Cannabis sativa*, *Lespedeza cuneata*, *Plantago major*). Notably, high antiparasitic efficacy in *Eupatorium altissimum* and *Abutilon theophrasti* is novel findings. Furthermore, efficacious species including *Dalea purpurea, Cichorium intybus, Lotus corniculatus,* and *Onobrychis viciifolia*, exhibited favorable nutritional profiles (low fiber, high digestibility), suggesting their properties as dual-purpose forages that are both nutritious and antiparasitic. Moreover, the high antiparasitic efficacy given by aqueous extractions underscores the practical potential of these plants to be applied on farms without elaborate processing. Collectively, these findings provide a foundation for *in vivo* validation and support the future development of integrated parasite management strategies for small ruminant production.

## Data Availability

The original contributions presented in the study are included in the article/supplementary material, further inquiries can be directed to the corresponding author.
