# Correction: Larvicidal activity of antiparasitic plant extracts against ovine gastrointestinal nematodes: an *in vitro* study

**DOI:** 10.3389/fvets.2026.1901461

**Published:** 2026-07-14

**Authors:** Chao Ke, Niya Tu, Fatimat Shittu, Md Imranuzzaman, Dipsana Kc, Men Su, Thomas B. McFadden, Tumen Wuliji

**Affiliations:** 1 Division of Animal Science, College of Agriculture, Food and Natural Resources, University of Missouri, Columbia, MO, United States; 2 Department of Agriculture and Environmental Sciences, Lincoln University, Jefferson City, MO, United States

**Keywords:** anthelmintic resistance, anti-parasitic forage plants, *Haemonchus contortus*, larval motility assay, small ruminants

Author Niya Tu's name was incorrectly written as "Tu Niya" in the published article. The correct author list reads:

Chao Ke^1*^, Niya Tu^2^, Fatimat Shittu^2^, Md Imranuzzaman^2^, Dipsana Kc^2^, Men Su^1^, Thomas B. McFadden^1^ and Tumen Wuliji^2^

1. Division of Animal Science, College of Agriculture, Food and Natural Resources, University of Missouri, Columbia, MO, United States,

2. Department of Agriculture and Environmental Sciences, Lincoln University, Jefferson City, MO, United States

The Author Contributions Statement has been corrected to read:

CK: Visualization, Formal analysis, Validation, Data curation, Writing – review & editing, Conceptualization, Investigation, Methodology, Software, Writing – original draft. NT: Writing – review & editing, Methodology. FS: Writing – review & editing, Methodology, Data curation. MI: Methodology, Writing – review & editing. DK: Methodology, Writing – review & editing. MS: Methodology, Writing – review & editing. TM: Visualization, Data curation, Writing – review & editing, Validation, Methodology, Conceptualization, Supervision. TW: Supervision, Investigation, Conceptualization, Writing – review & editing, Resources, Validation, Funding acquisition, Project administration, Methodology.

An incorrect Funding statement was provided. The funder “The 1890 Universities Foundation's Center of Excellence in Land Water and Resource Management”, FY22-NREE-Lincoln-37307- Grant Fund to TW, was erroneously omitted. The correct funding statement reads:

## Funding

The author(s) declared that financial support was received for this work and/or its publication. This study was supported by the USDA-NIFA, project award no. 2021-38821-34787 from the U.S Department of Agriculture's National Institute of Food and Agriculture to Lincoln University. Wuliji: Sustainable Gastrointestinal parasite control in small Ruminants. The 1890 Universities Foundation's Center of Excellence in Land Water and Resource Management has sponsored publication of this article from FY22-NREE-Lincoln-37307- Grant Fund to TW: Small Ruminant Grazing Prescription for Under-story Forage, Weed, and Invasive Plant Management on Woodland Pasture Systems.

The original version of this article has been updated.

